# Analytical and Clinical Validation of a Digital Sequencing Panel for Quantitative, Highly Accurate Evaluation of Cell-Free Circulating Tumor DNA

**DOI:** 10.1371/journal.pone.0140712

**Published:** 2015-10-16

**Authors:** Richard B. Lanman, Stefanie A. Mortimer, Oliver A. Zill, Dragan Sebisanovic, Rene Lopez, Sibel Blau, Eric A. Collisson, Stephen G. Divers, Dave S. B. Hoon, E. Scott Kopetz, Jeeyun Lee, Petros G. Nikolinakos, Arthur M. Baca, Bahram G. Kermani, Helmy Eltoukhy, AmirAli Talasaz

**Affiliations:** 1 Department of Medical Affairs, Guardant Health, Inc., Redwood City, California, United States of America; 2 Department of Research and Bioinformatics, Guardant Health, Inc., Redwood City, California, United States of America; 3 Rainier Hematology Oncology, Northwest Medical Specialties, Puyallup, Washington, United States of America; 4 Department of Medicine, University of California San Francisco School of Medicine, San Francisco, California, United States of America; 5 Genesis Cancer Center, Hot Springs, Arkansas, United States of America; 6 Department of Molecular Oncology, John Wayne Cancer Institute at Saint John's Health Center, Santa Monica, California, United States of America; 7 Department of Gastrointestinal Medical Oncology, The University of Texas MD Anderson Cancer Center, Houston, Texas, United States of America; 8 Department of Medicine, Division of Hematology-Oncology, Samsung Medical Center, Sungkyunkwan University School of Medicine, Seoul, Korea; 9 Department of Hematology and Medical Oncology, University Cancer and Blood Center, Athens, Georgia, United States of America; 10 Administration, Guardant Health, Inc., Redwood City, California, United States of America; Deutsches Krebsforschungszentrum, GERMANY

## Abstract

Next-generation sequencing of cell-free circulating solid tumor DNA addresses two challenges in contemporary cancer care. First this method of massively parallel and deep sequencing enables assessment of a comprehensive panel of genomic targets from a single sample, and second, it obviates the need for repeat invasive tissue biopsies. Digital Sequencing^TM^ is a novel method for high-quality sequencing of circulating tumor DNA simultaneously across a comprehensive panel of over 50 cancer-related genes with a simple blood test. Here we report the analytic and clinical validation of the gene panel. Analytic sensitivity down to 0.1% mutant allele fraction is demonstrated via serial dilution studies of known samples. Near-perfect analytic specificity (> 99.9999%) enables complete coverage of many genes without the false positives typically seen with traditional sequencing assays at mutant allele frequencies or fractions below 5%. We compared digital sequencing of plasma-derived cell-free DNA to tissue-based sequencing on 165 consecutive matched samples from five outside centers in patients with stage III-IV solid tumor cancers. Clinical sensitivity of plasma-derived NGS was 85.0%, comparable to 80.7% sensitivity for tissue. The assay success rate on 1,000 consecutive samples in clinical practice was 99.8%. Digital sequencing of plasma-derived DNA is indicated in advanced cancer patients to prevent repeated invasive biopsies when the initial biopsy is inadequate, unobtainable for genomic testing, or uninformative, or when the patient’s cancer has progressed despite treatment. Its clinical utility is derived from reduction in the costs, complications and delays associated with invasive tissue biopsies for genomic testing.

## Introduction

Next-generation sequencing (NGS) of solid tumor tissue has advanced our ability to perform multiplex testing for an ever-growing number of targetable genomic alterations utilizing small needle biopsies or archival pathology slides. As the number of genomic targets with matched therapies grows, tissue biopsy material can be quickly exhausted before multiple “companion diagnostics” tests can be completed. For example, the National Comprehensive Cancer Network (NCCN) guidelines for non-small cell lung cancer (NSCLC) recommend measurement of genomic alterations in seven different genes (*EGFR*, *ALK*, *ERBB2* (encodes HER2 protein), *BRAF*, *MET*, *ROS1* and *RET*) to guide twelve different matched therapies [1]. Because serial singleplex testing for all these genomic alterations poses a challenge when tissue biopsy samples are limited, the NCCN NSCLC guidelines now recommend NGS testing as an efficient means of multiplex testing on a small tissue sample [1]. In addition to lung cancer, NCCN guidelines currently recommend matching of genomic targets to targeted therapies as a first line therapy strategy in many additional cancers, including chronic myelogenous leukemia, metastatic melanoma, breast cancer, gastric cancer, gastrointestinal stromal tumor, hepatocellular carcinoma and colorectal cancer [2]. At the same time, tissue-based NGS has several practical challenges: biopsy samples may be of insufficient quantity or unavailable/unobtainable, initial biopsies may not reflect the current status of a tumor’s genetic profile over time, and small biopsies using needle aspiration or microforceps may not capture inter- and intra-tumor heterogeneity in the same patient [3].

Since most metastatic cancers are unresectable, genomic testing frequently relies on relatively small core or fine needle aspiration or microforceps biopsies [4]. In 2012 the UK National Lung Cancer Audit Report found that 23% of lung biopsies had insufficient material for cyto- or histo-pathological diagnosis, let alone molecular diagnosis [5]. Even when small biopsies are sufficient for pathological diagnosis and stains, the remaining tissue may be “quantity not sufficient” (QNS) for genomic analysis. In 2010, the International Working Group on Multidisciplinary Lung Adenocarcinoma Classification estimated that only 57% of such biopsies had sufficient tissue for genomic analysis after initial pathology diagnosis and staining [6,7]. In the Lung Cancer Mutation Consortium study of 14 U.S. academic centers, one oncogenic driver mutation could be tested in 91% of tissue specimens but the ten desired target gene alterations could only be measured in 66% of them, a shortfall of 25% [8]. In a study of advanced breast cancer, yield for molecular testing was even lower (36%) for biopsy samples from bone, the most common site of breast cancer metastasis [9]. When tissue biopsies have insufficient tumor cell content or are QNS for genomic analysis, formalin-fixed paraffin embedded (FFPE) material or slides for tissue-based NGS are often unavailable or unobtainable, or may be outdated because they no longer reflect the current genomic status of the tumor [10].

Intra- or inter-tumor heterogeneity may limit the sensitivity of tissue-based NGS for genomic alterations. Tumor sampling errors can occur because of genomically different sub-clones within the same primary tumor, between the primary tumor and its metastases, or between metastatic lesions [11,12]. In a tissue-based NGS study of two renal cell carcinomas, nineteen different biopsies of the two tumors each typically missed one-third of the nonsynonymous somatic mutations found elsewhere in the tumor despite sequencing to 250-fold coverage [13]. Considerable tumor heterogeneity has now been established in numerous other major cancers and appears to be the rule rather than the exception [14], posing the predicament that needle, forceps or even surgical biopsies may not capture all the various clones/genomic alterations relevant to targeted therapy.

Since the discoveries of circulating cell-free DNA (cfDNA) by Mandel and Metais in 1948 and of cell-free circulating tumor DNA (ctDNA) by Stroun in 1987, technologies to non-invasively interrogate individual cancer genomes as an alternative to invasive tissue biopsy have undergone gradual and steady improvement [15–17]. Cell-free circulating tumor DNA consists mainly of 166 base pair double-stranded DNA fragments resulting from apoptosis, necrosis or release of nuclear DNA into the circulation [18,19]. Because these fragments have a short half-life of 1.5 hours in circulation due to rapid hepatic and renal clearance, ctDNA reflects a real-time genomic signature of the tumor versus NGS of archival tissues which may only reflect the cancer’s status at an earlier static time point [20,21]. DNA isolated from cancer tissue or fetal DNA isolated from maternal blood are often available in relatively high mutant allele fractions (MAFs) of 2%-10% relative to ctDNA which may be present at concentrations an order of magnitude lower [22]. The key challenge to NGS of ctDNA is that at such low concentrations of mutated DNA fragments the signal is obscured by the noise inherent in even the most advanced sequencing machines. Thus, dramatic improvements in specificity at low DNA inputs are required in order to move beyond “hot-spot” testing towards sequencing complete exons in the several dozen gene targets for matched therapies.

Guardant360 is an NGS panel of 54 clinically actionable genes (S1 Table) utilizing digital sequencing of cell-free circulating tumor DNA isolated from a simple, non-invasive blood draw. Its key differentiating characteristic from other “liquid biopsy” methods is its ultra-high specificity. Digital sequencing (see Materials and Methods) employs pre-sequencing preparation of a digital library of individually tagged cfDNA molecules combined with post-sequencing bioinformatic reconstruction to eliminate nearly all false positives. The test detectes single nucleotide variants in all 54 genes and copy number amplifications in *EGFR*, *ERBB2* (codes for HER2) and *MET* [23]. This test is indicated to prevent repeat invasive biopsy when an initial biopsy is QNS or otherwise unavailable/unobtainable as well as when cancer has progressed or recurred despite treatment. Actionable genomic alterations are those for which there is an FDA-approved treatment or that serve as eligibility criteria for later phase clinical trials. Guardant360 is an advanced diagnostic laboratory test offered by a sole source laboratory certified by the Clinical Laboratory Improvement Amendments (CLIA) for high complexity (molecular pathology) testing and accredited by the College of American Pathology (CAP).

Due to high rates of false positives with traditional NGS assays when tumor DNA is in low concentrations, the majority of “liquid biopsy” methods interrogating cell-free DNA have been limited to hotspot analyses, amplicon sequencing approaches and/or typically involve patients where the ctDNA fraction is greater than 1–5% of total circulating cfDNA [24,25]. Since the numbers of false positives increase as the targeted region for sequencing increases, these assays manage the false positive rate by limiting the sequenced region to a very short “hotspot” or limited number of hot exons. However, it has been shown with tissue-based NGS that many actionable genomic alterations may be missed when analysis is limited to hotspots or even “hot” exons [26]. In addition to our technology, several other promising approaches are emerging to solve this problem [27–29].

Here we report the analytical and clinical validation of the 54-gene (512 exons) Guardant360 panel of mutations and gene amplifications in conformance with evidentiary standards established by the Standards for Reporting of Diagnostic Accuracy (STARD), Evaluation of Genomic Applications in Practice and Prevention (EGAPP), NCCN Task Force recommendations and the recent Next-generation Sequencing: Standardization of Clinical Testing (Nex-StoCT) biomarker guidelines [30–33]. In a comparison to whole exome sequencing, digital sequencing sets a new gold standard for specificity, with no false positive mutation calls in almost 1.6 million bases sequenced. This diagnostic performance is critical to the measurement of a comprehensive panel of genomic alterations at the very low concentrations typical of circulating tumor DNA. With high sensitivity and the ultra-high specificity required to comprehensively sequence cfDNA, the Guardant360 test has the potential to evaluate the multiple genomic alterations cited in NCCN guidelines, to act as a “summary” of the different tumor clones in patients with tumor heterogeneity [34], to interrogate all actionable genomic alterations via comprehensive sequencing of a multigene panel, and to prevent the time delays, cost and complications inherent in invasive biopsies. Actual experience testing the first 1,000 consecutive patients in clinical practice found an assay failure rate of 0.2%. Although the 54-gene panel is not as comprehensive as some 300–400 gene tissue-based NGS panels, an actionable result was produced in over 3/4 of the advanced cancer patients with a positive finding within the 54-gene panel.

## Results

**Fig 1:**
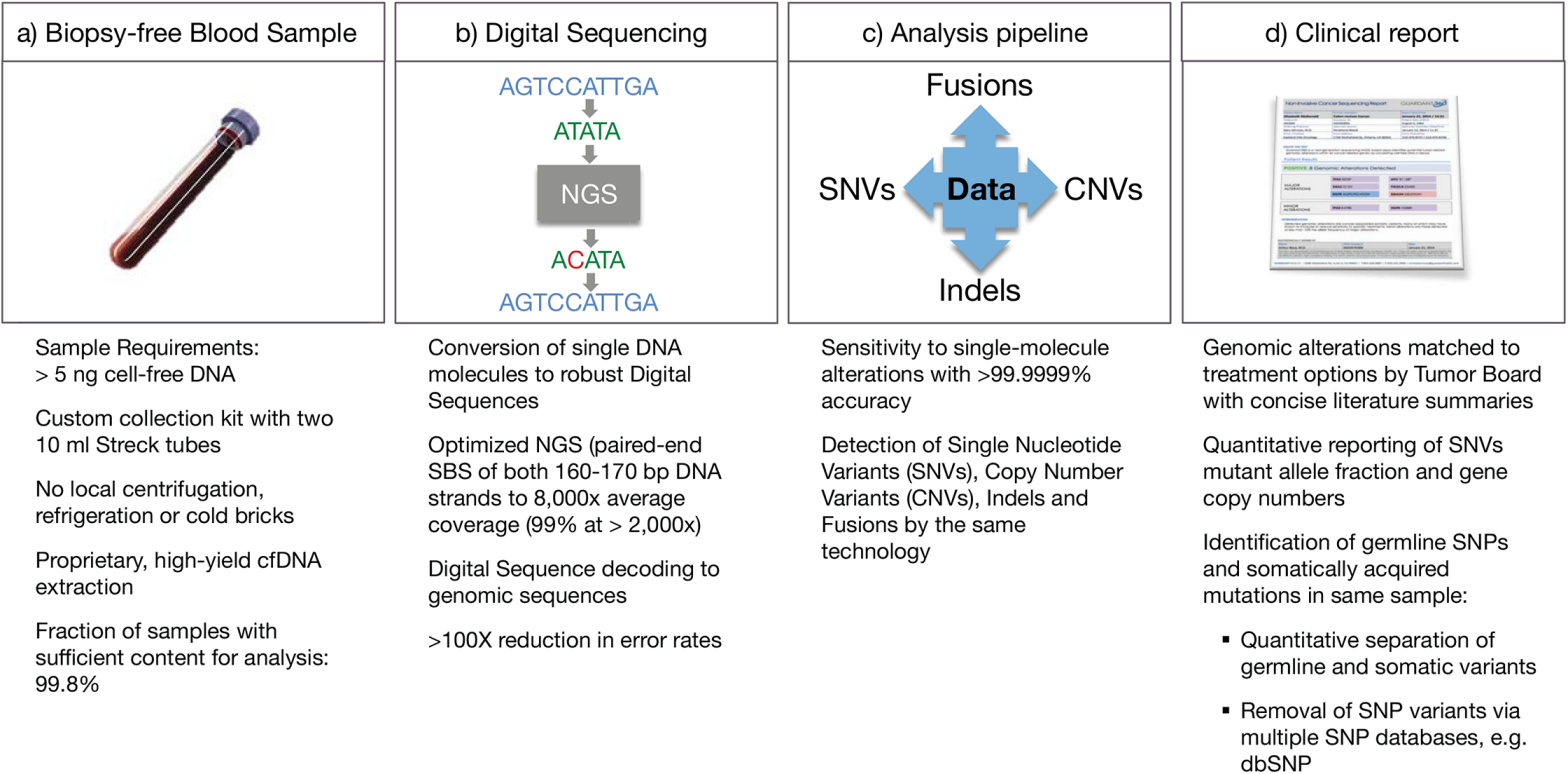
Workflow for the Guardant360 cell-free circulating DNA NGS genomic profile. (a) cfDNA is extracted from a routine blood draw. (b) 5.0–30 ng of DNA undergoes digital library preparation including oligonucleotide barcoding of each strand in each individual DNA fragment. Complete sequencing of 512 exons in 54 cancer-related genes is conducted with the HiSeq 2500 (Illumina). Multi-analyte algorithms and bioinformatics are used to reconstruct the progenitor cfDNA fragment sequences without false positives. (c) Sequence data are processed using a customized analysis pipeline designed to accurately detect the four major classes of genomic alterations. (d) Mutant allele fractions are reported quantitatively for somatic single nucleotide variants of clinical significance and distinguished from germline single nucleotide variants (SNVs) by reference to the COSMIC and dbSNP databases, as well as their concentrations.

The Guardant360 cfDNA assay workflow is illustrated in Fig 1.

### Digital sequencing methodology vs. traditional NGS

**Fig 2:**
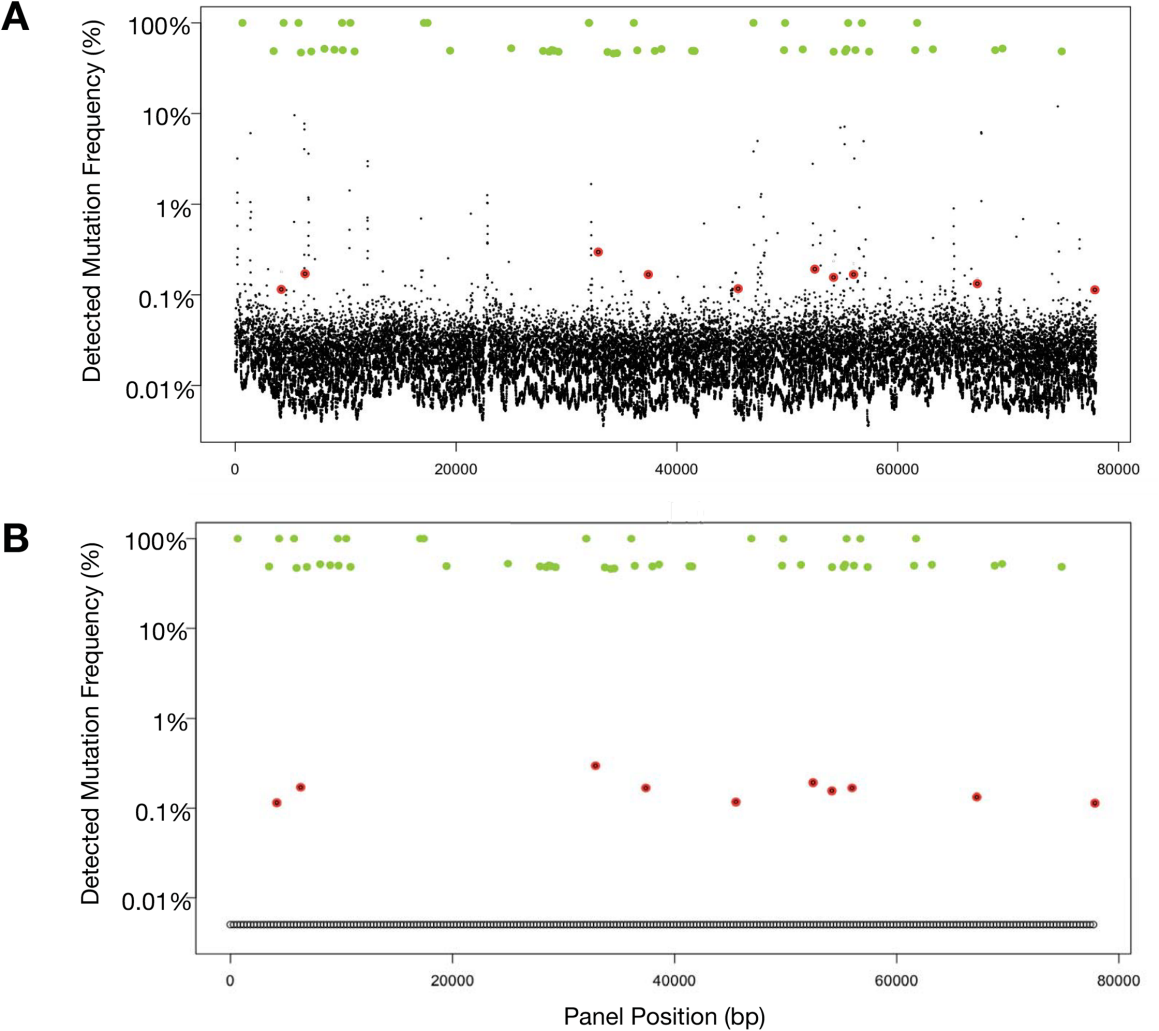
Fig 2A illustrates output from Illumina HiSeq using standard library prep on cell-free DNA sample spiked with samples from ten cell lines with known single nucleotide variant (SNV) mutations. Germline single nucleotide polymorphisms (SNPs) (green dots) at either 50% (heterozygous) or 100% (homozygous) mutant allele fractions (MAF). In contrast, the ten somatic SNVs (red dots) are quantitated at much lower MAF typically encountered with cell-free circulating tumor DNA, and are obscured by the false positive “noise” associated with low DNA concentrations. The larger the targeted region, the more false positive signals are encountered. In this actual sample, sequencing the long targeted region (78 kbp) required for the 54-gene panel results in 224 false positives at the 0.1% to 10% MAFs, making accurate sequencing of ctDNA unworkable. Fig 2B utilizes the same sample as in Fig 2A but was analyzed with Digital Sequencing technology. Molecular techniques in the pre-analytic/pre-sequencing phase and bioinformatics in the post-sequencing phase are employed to eliminate the “noise” in a process analogous to the signal transduction processing-enabled conversion of analog voice and television signals to digital signals. The result is sensitivity to the level of 1–2 mutated DNA fragment molecules in up to 1,000 wild type (mostly leukocyte-derived) DNA fragments overlapping the same nucleotide base position, essentially eliminating the false positives normally encountered at low MAFs when sequencing large targeted regions.

We compared the performance of digital sequencing to traditional next-generation sequencing using TruSeq DNA Sample Preparation and bioinformatics pipelines with removal of low quality reads (i.e. Qscore < 30). When cancer cell-line cfDNA with ten known mutations are spiked at 0.1% MAFs into a background of cfDNA extracted from a healthy donor and sequenced with an Illumina HiSeq 2500 with standard prep, these SNVs are obscured by a plethora of false positives with MAFs of 0.05–5% (Fig 2A). Digital sequencing eliminates these false positives (as illustrated in Fig 2B) enabling high-quality sequencing of each single molecule of cfDNA. These figures also illustrate that both germline and tumor-derived SNVs are analyzed and quantitated simultaneously. SNV allele fractions around 50% or 100% generally (when tumor fraction in circulation is low) may be used to identify heterozygous or homozygous single nucleotide polymorphisms (SNPs), respectively.

### Analytic specificity and sensitivity

**Fig 3:**
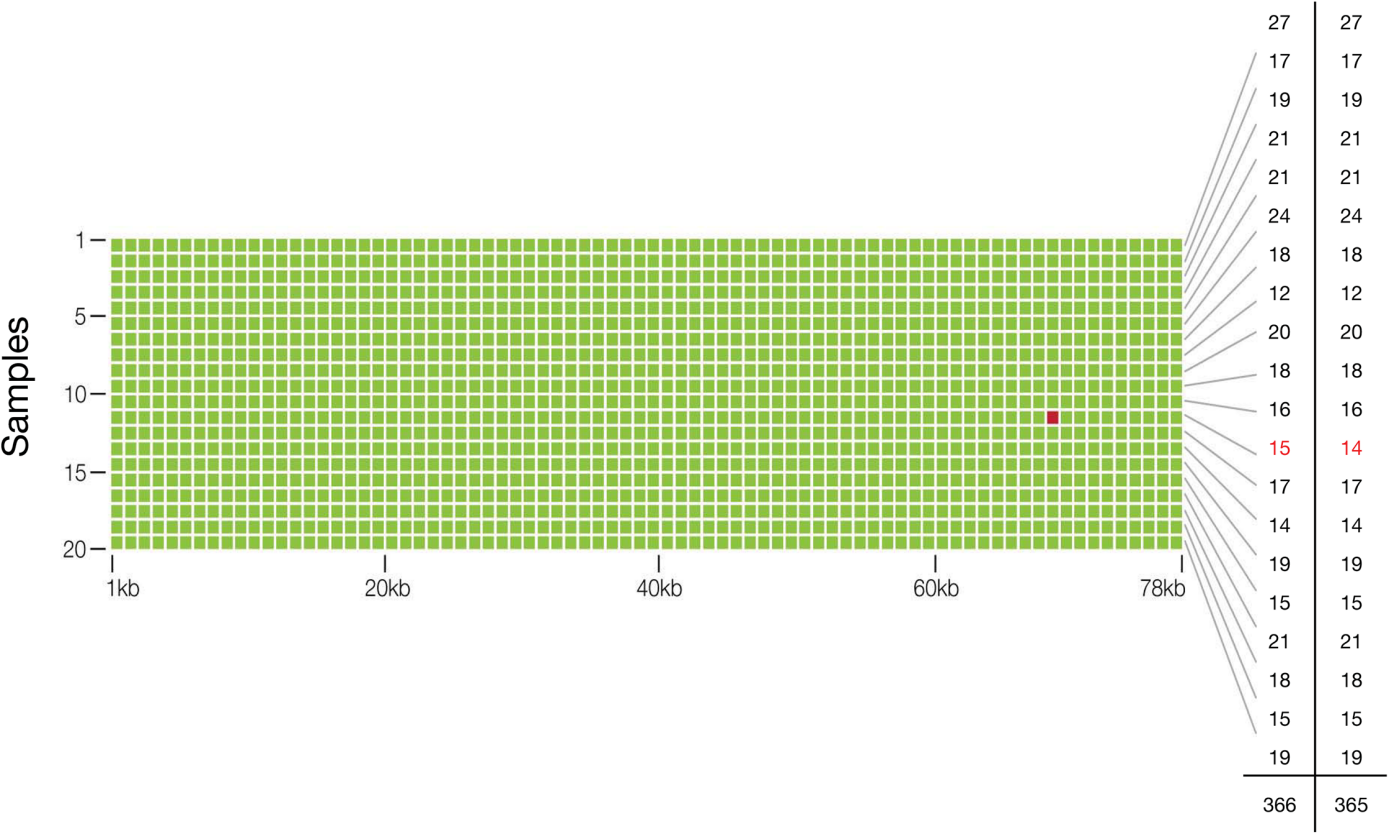
Analytic Specificity and Sensitivity, and Diagnostic Accuracy of the Guardant360 cell-free circulating tumor DNA assay. The matrix illustrates that a targeted region of 78 kbp was sequenced in each of 20 samples to identify single nucleotide variants at approximately 2.0–5.0% MAF (heterozygous) in circulating cfDNA. Green dots represent concordant single nucleotide variant (SNV) calls for digital sequencing of 10 ng cfDNA to calls made by whole exome sequencing of 2 μg of genomic (leukocyte) DNA from the same sample. The red dot represents the single false positive result in a cumulative 1.56 million bases sequenced. The far right-hand column illustrates the number of SNVs per sample identified with exome sequencing, and the penultimate column with digital sequencing, for a total of 365 and 366 SNVs, respectively.

To assess the accuracy of our test, two micrograms of genomic DNA from 20 healthy and young donors (AllCells, Alameda, CA) were sent to an outside reference lab (Ambry Genetics, Aliso Viejo, CA) for whole exome sequencing. Ten nanograms of matched cfDNA samples were spiked at 5% (actual range observed was 4–10%, or 2–5% if for a heterozygous SNV) into another cfDNA sample and processed using Guardant360. Across the 78,000 base pair (78 kbp) panel, the whole-exome sequencing assay found 365 SNVs (mean 18 SNPs (range 12–27) per sample) and Guardant360 identified all 365 SNVs with one additional false positive SNV relative to whole exome sequencing (Fig 3). Thus analytic sensitivity for SNVs at 2% to 10% MAFs was 100%. The finding of 365 true positives and a single false positive in a cumulative targeted region of 1.56 million base pairs (20 samples x 78 kbp per sample), equates to analytic specificity of 99.9999%, or a 0.0001% false positive rate (Table 1).

**Table 1:** Two by two table comparison of the diagnostic performance of digital sequencing to whole exome sequencing of the 54-gene panel. Twenty samples at 78 kpbs per sample were sequenced with a single apparent false positive and no false negatives, resulting in 100% analytic sensitivity (at roughly 2.5% SNV mutant allele frequencies) and near-perfect 99.9999% specificity.

	Reference (Whole Exome Sequencing)		
	Positive	Negative	
Guardant360 (Digital Sequencing)			Total
Positive	365	1	366
Negative	0	1,559,634	1,559,634
**Total**	365	1,559,635	1,560,000
	**Diagnostic Accuracy**	99.9999% (95% CI 99.9996%–99.9999%)	
	**Analytic Sensitivity**	100% (95% CI 98.9944%–100%)	
	**Analytic Specificity**	99.9999% (95% CI 99.9996%–99.9999%)	

Since whole exome sequencing was used as the reference standard and Ambry reports 99% accuracy using microgram DNA input amounts, the false positive found via digital sequencing could have been a false negative via whole exome sequencing. To determine the true germline nucleotide identity of position chr11:534,242 (*HRAS*) in the putative false positive sample, genomic leukocyte DNA (gDNA) was extracted from blood and sent for Sanger sequencing (Stanford Protein and Nucleic Acid facility). All sequencing traces show an equal intensity peak corresponding to A and G nucleotides at this position indicating a heterozygous germline SNP in *HRAS* (H27H), consistent with the digital sequencing data. Thus, the nucleotide call using digital sequencing was correct, indicating no false positives in the approximately 1.56 million base pairs sequenced and the analytic specificity of digital sequencing was resolved as > 99.9999%.

**Fig 4:**
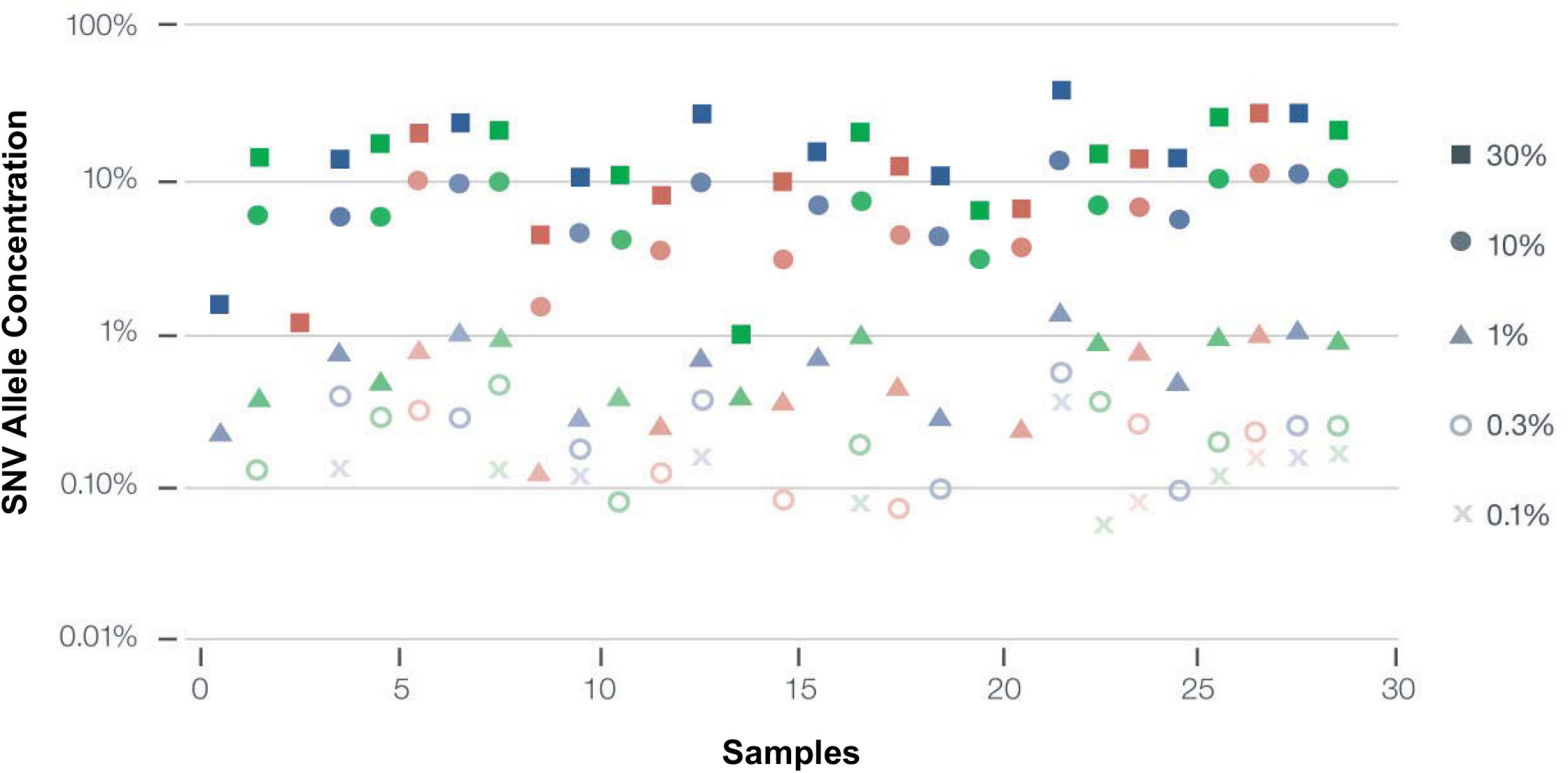
Analytic Sensitivity of the Guardant360 digital sequencing method. Twenty nine SNV samples were diluted serially until they could no longer be measured with the assay. Each column represents successively greater serial dilutions of a given sample. The limit of detection (LOD) was 0.25% mutant allele frequency or fraction) (MAF), defined as the percentage at which > 80% of samples were detected. Note that almost 30% of samples were additionally detected at 0.1% MAF or lower, where 0.1% represents a single mutated DNA fragment out of 999 wild-type (leukocyte-derived) DNA fragments overlapping the same nucleotide base.

Analytic sensitivity based on serial dilutions of 29 different SNVs was conducted in triplicate. The SNV fraction at which > 80% of SNVs are detected is defined as the limit of detection and was 0.25% (Fig 4). In 28% of the samples, dilution to 0.1% or lower could still be detected. This corresponds to one DNA fragment with a SNV at a given nucleotide base position divided by 999 wild-type DNA fragments overlapping the same base position.

As expected, the accuracy of quantification decreases from SNVs at high MAF to low MAF. For example, the absolute error for a high MAF of 10% would be ± 0.4%, while an SNV at 1% MAF would have a relatively greater absolute error (relative to its 1% MAF) of ± 0.2% (Table 2).

**Table 2:** Mutant Allele Frequency measurement accuracy. Quantification uncertainty varies inversely with single nucleotide variant MAF, with the highest relative uncertainty at lower MAF and least percent relative uncertainty at higher MAF.

MAF range (%)	Quantification Uncertainty % (Absolute Error)
> 10	4% (> 0.4)
3–10	6% (0.18–0.6)
1–3	14% (0.14–0.42)
0.5–1.0	20% (0.1–0.2)
0.25–0.5	30% (0.075–0.15)

To determine repeatability of the Guardant360 test, replicate sequencing was repeated twice for 5 of the samples used in the accuracy study with the same lot, operator and sequencer with 100% concordance between the replicates (within run precision). Similarly, to determine reproducibility, replicate sequencing was repeated twice for 5 of the samples used in the accuracy study with different reagent lots, different operator and at least one week following initiation of the experiment, with 100% concordance between the replicates (between run precision).

### Assay robustness/interference from genomic DNA secondary to storage time and temperature variation

**Fig 5:**
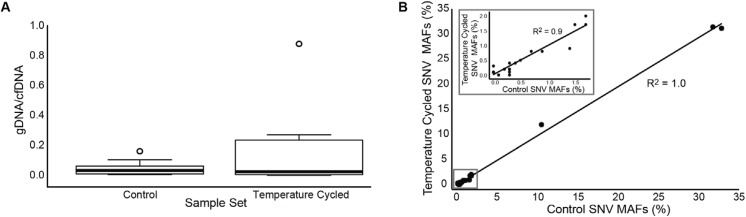
Guardant360 samples stressed by prolonged storage time and high temperature cycling do not impact performance. A) Extent of genomic DNA (gDNA from leukocytes) contamination in the control cell-free DNA (cfDNA) samples compared to prolonged temperature cycled samples sets represented as the ratio of gDNA (> 500 bps) to cfDNA (< 500 bps). Temperature cycled samples were incubated at 37°C for 8 hours followed by 16 hours at room temperature daily for 5 days. Control samples were processed immediately upon receipt at the laboratory. Center lines show the medians; box limits indicate the 25^th^ and 75^th^ percentiles as determined by R software; whiskers extend 1.5 times the interquartile range from the 25^th^ and 75^th^ percentiles, outliers are represented by dots. N = 10 sample points. B) Concordance of control sample set with prolonged temperature cycled sample set shown by a correlation plot of all mutant allele frequencies (MAFs) of the single nucleotide variants (SNVs) from the control sample set versus the corresponding SNV MAFs in the temperature cycled sample set. The total number of different SNVs plotted is 24. The inset shows the correlation of MAFs at less than or equal to 2% between the two data sets, showing excellent correlation even when SNVs are as low as 2.5%.

Assay robustness was evaluated by heating 10 plasma samples to 37°C for eight hours per day for a total of five days. The extent of gDNA contamination was determined semi-quantitatively by analysis with the Agilent 2100 bioanalyzer and only 3 of 10 demonstrated contamination with genomic DNA, of which 2 samples demonstrated low levels of gDNA and one sample exhibited a moderate amount of contamination. Next, these samples were compared to 10 split sample controls processed with no time delay upon receipt or temperature cycling (after two days shipping). The MAFs for the 24 SNVs in the paired ten samples were plotted in a correlation plot with R^2^ = 1.0 (Fig 5). Concordance was 100% between tubes for all SNVs with MAFs above 0.3% and ~90% for MAFs > 0.1% (Fig 5).

### Assessment of copy number variation (CNV)

CNVs for three genes, *EGFR*, *ERBB2* and *MET*, were determined by utilizing nine cell lines well-characterized by fluorescence in situ hybridization (FISH) [35–39]. The cfDNA from these cell lines was extracted from cell culture media and sequenced from libraries prepared using 15 ng input amounts over two separate flow cells. Results showed an excellent correlation with high diagnostic accuracy (Table 3).

**Table 3:** Guardant360 gene copy number validation results. Low/High levels of amplification refer to 3–4 or >9 copy numbers, respectively.

Cell Line	Gene	Expected Level of Amplification	Guardant360 Absolute Gene Copy Number	Concordant? (Y/N)
A431	*EGFR*	High	11.94	Y
AU565	*ERBB2*	High	17.92	Y
BT474	*ERBB2*	High	15.14	Y
CAL27	*EGFR*	Low	3.76	Y
HCC827	*EGFR*	High	36.85	Y
HCC2218	*ERBB2*	High	16.32	Y
NCI-H1648	*MET*	High	8.19	Y
NCI-H1993	*MET*	High	30.29	Y
NCI-H2009	*MET*	Low	2.87	Y

Within-run (repeatability) and between-run (reproducibility) precision portion of the validation study were shown to be > 90% concordant between different runs, operators and reagents for the three gene amplifications each run in three replicates and in three separate runs. No false positive results were noted in any of the CNV studies. Through 6 separate titration/serial dilution experiments using 3 different cfDNA amounts in duplicate, limit of detection (LOD) for CNVs in *EGFR*, *ERBB2* and *MET* was determined to be 0.2, 0.5 and 0.2 extra copies, respectively. Because a highly amplified gene is easier to detect, the results can be explained as corresponding to detection of low level (copy number = 6) *MET* and *EGFR* amplification in patients with ~5% tumor fraction or detection of high-level (copy number = 10) amplification in patients with ≤2% tumor fraction. For *ERBB2*, this corresponds to detection of low-level amplification in patients with ~12% tumor fraction or detection of high-level amplification in patients with ≤6% tumor fraction (S1 Fig).

### Observational data from 510 patient multicenter study and 1,000 consecutive samples in clinical practice

510 plasma samples from stage III/IV patients with a broad range of non-hematologic malignancies from five different centers were analyzed via digital sequencing. 504 samples (98.8%) were run successfully. The average number of genomic alterations was 3.3 (range 0–120) and 86% of samples were positive for a genomic alteration. The range of MAF was 0.1% to 94.6%, the latter in a stage IV breast cancer patient who succumbed two weeks later (Table 4).

**Table 4:** Observational results from 510 patient multicenter research study utilizing the Guardant360 54-gene panel.

Number of passed samples	504 (98.8%)
Mutant allele fraction median (range)	0.6% (0.1%–94.6%)
Percent of samples with at least one somatic alteration	86%
Mean alterations per positive sample (range)	3.3 (1–120)

Results from the first 1,000 consecutive samples processed in the CLIA laboratory included only two samples failing quality control (one for inability to separate plasma from the buffy coat despite multiple ultracentrifugation steps and the other for inadequate cfDNA quantity < 5.0 ng). All 998 remaining samples were successfully sequenced for a 99.8% assay success rate. Final results could not be reported for 4 patients (two had fetal DNA and two had transplant donor DNA present). The patients had a broad range of non-hematologic malignancies (excluding primary brain tumors) and although stage was often unascertainable, some stage II patients were tested. The average number of genomic alterations was 3.1 (range 0–44) and 76.2% of samples were positive for a genomic alteration. Overall, 76.1% of genomic alterations found were actionable: 11.7% had an FDA approved matched therapy for the specific mutation and cancer type, 52.1% had an approved matched therapy for the specific mutation but in a different cancer type from the patient, and 76.1% of patients had a genomic alteration that was an eligibility criterion for a clinical trial (Table 5).

**Table 5:** Initial 1,000 consecutive samples from clinical practice (pan-cancer stages III-IV).

Number of passed samples	998 (99.8%)
Mutant allele fraction median (range)	0.5% (0.1%–75.1%)
Percent of samples with at least one somatic alteration	76%
Mean alterations per positive sample (range)	3.1 (1–44)
Percent of positive samples actionable	76%

**Fig 6:**
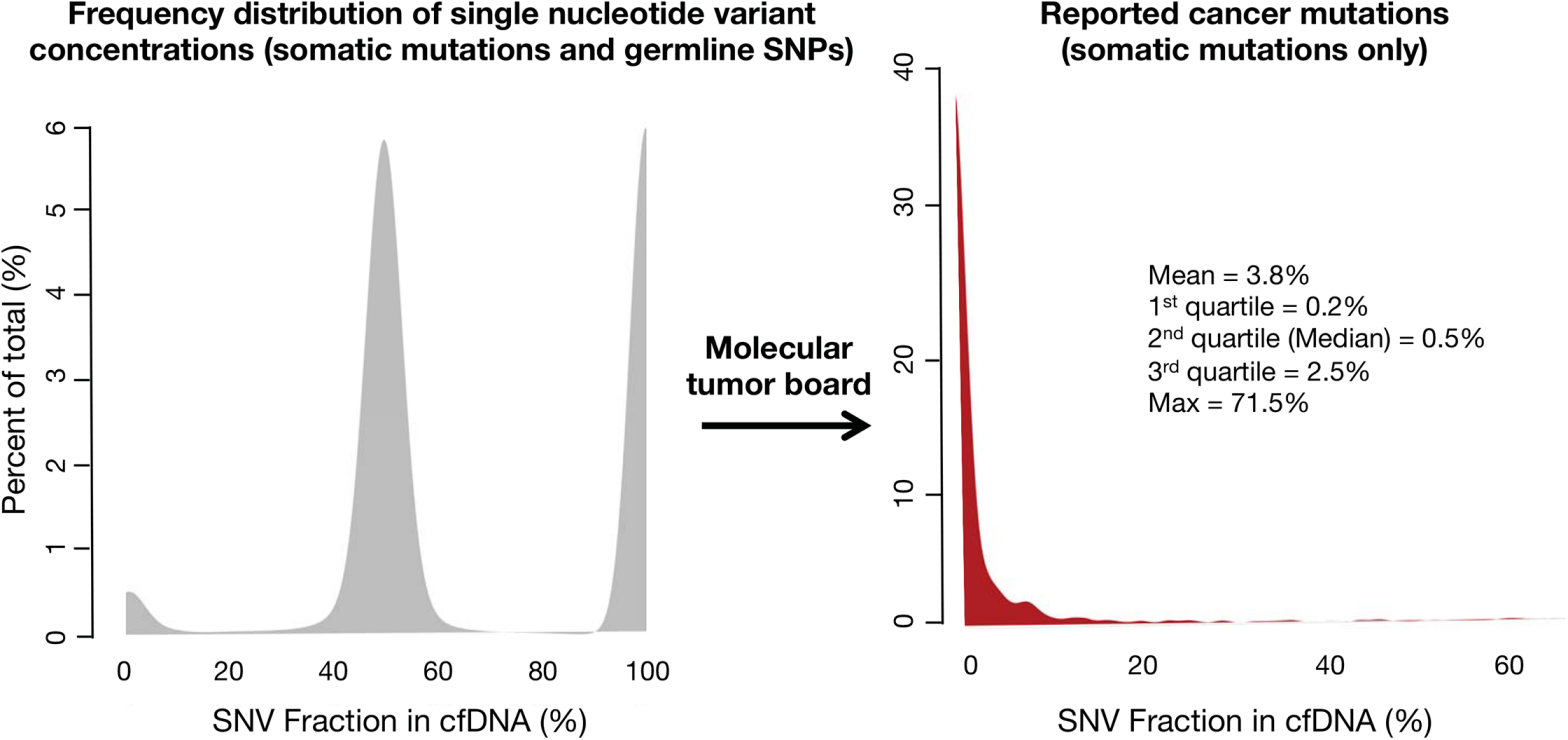
Frequency distribution of single nucleotide variant (SNV) mutant allele fractions (MAFs) for the first 1,000 consecutive patients tested in clinical practice (broad range of non-hematologic malignancies). On the left the three peaks represent kernel distribution density plots of the frequencies of somatic and germline mutant allele fractions. By their low concentrations, somatic mutations (far left-hand small peak) can be generally distinguished from heterozygous germline SNVs around 50% MAF (middle peak) and homozygous germline SNVs around 100% MAF (right-hand peak). Cell-free DNA is both leukocyte-derived and tumor-derived, with the germline DNA generally representing the bulk of the cfDNA. On the Guardant360 panel the molecular tumor board filters out the germline SNVs and only the somatic mutations are reported. The red curve in the right-hand figure shows the frequency distribution of the MAFs for the somatic SNVs only. The long tail reflects the rare patients with a somatic mutation at high MAF.

The frequency of distribution for germline SNPs and somatic SNVs is illustrated in Fig 6. The median (50^th^ percentile) MAF for somatic mutations was 0.5%, with 1^st^ quartile = 0.2%, 3^rd^ quartile = 2.5% and maximum value = 71.5%. The long tail of higher MAFs resulted in a mean = 3.8%.

### Analysis of control samples from healthy persons

During the technology development process, 79 healthy normal controls (source: AllCells) were tested and, in those, a single *TP53* R248Q mutation (heavy smoker, but no history of cancer) was observed, typical of a somatic mutation. During patient testing, normal controls are run with every sequencing batch resulting in an additional 143 healthy persons analyzed as controls. None of these individuals had a detectable somatic mutation in the 54-gene panel (single nucleotide polymorphisms (SNPs) were commonly seen but these are ascertained as germline SNPs because they occur at close to 50% or 100% mutant allele frequencies in cell-free DNA).

### Clinical validation of the cfDNA panel

**Fig 7:**
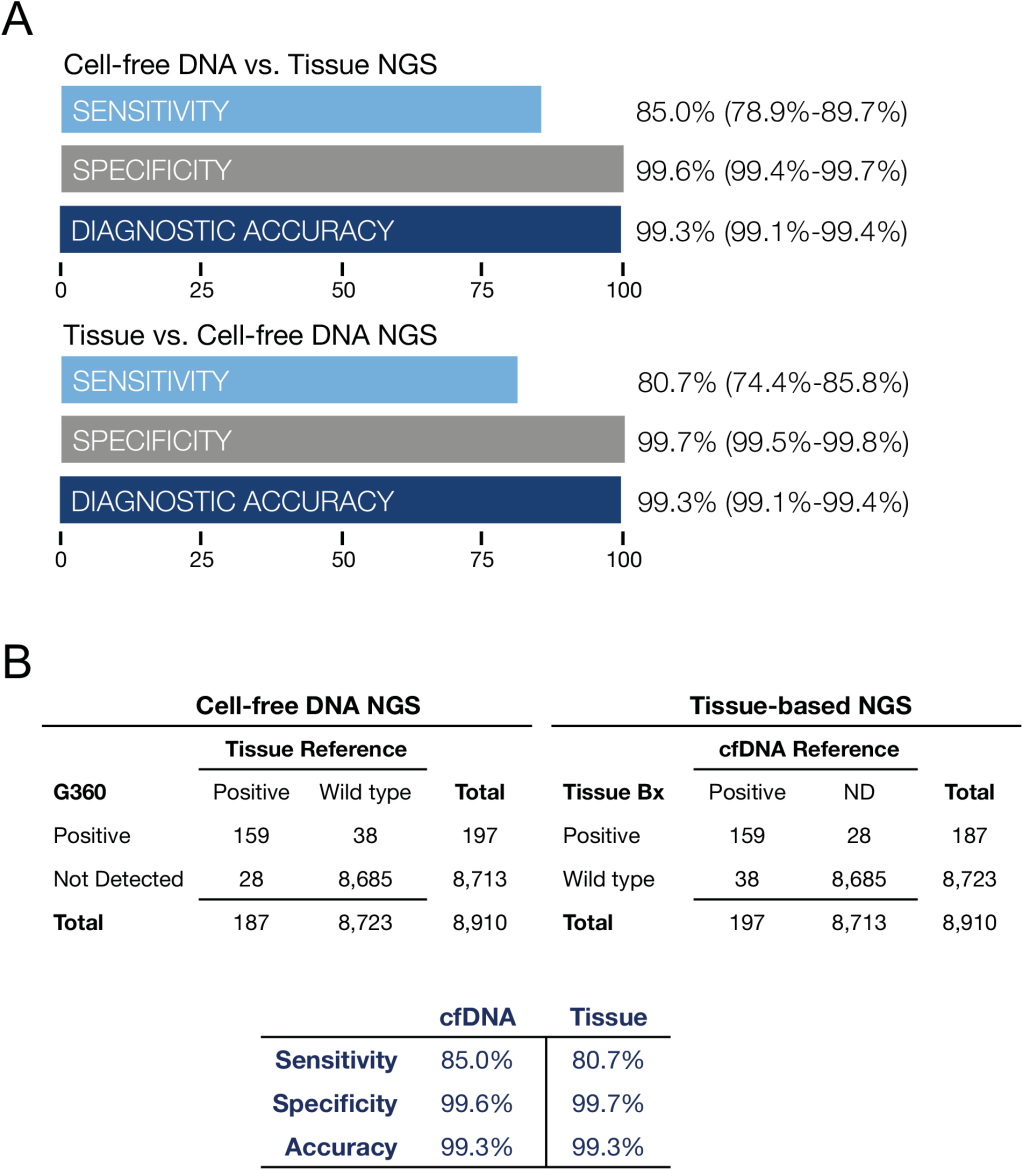
Fig 7A is a comparison of tissue NGS results biopsied at five outside institutions compared to cfDNA sequencing at Guardant Health on 165 paired plasma samples from stage III-IV solid tumor cancer patients. Data summarizes diagnostic test performance for all 54 mutated tumor suppressor and oncogenes. The most commonly mutated genes were *ALK, APC, BRAF, CDKN2A, CTNNB1, FBXW7, KRAS, NRAS, PIK3CA, PTEN*, and *TP53*. Sensitivity, specificity and diagnostic accuracy are shown with 95% confidence intervals.Fig 7B illustrates the two by two contingency tables corresponding to Fig 7A. On the left cfDNA NGS results are compared to tissue-based NGS as the reference standard. On the right tissue-based NGS results are compared to cfDNA findings as the reference standard. All gene mutations found in cfDNA and tissue DNA based on NGS of 54 genes are shown in S2 Table. Both methods demonstrate similarly high sensitivity and near-perfect specificity. For cfDNA, sensitivity is limited by the amount of tumor DNA shed into circulation and for tissue, sensitivity is likely limited by sampling error related to intra-or inter-tumor heterogeneity. The sampling error on tissue samples may be related to sub-sampling of tumor heterogeneity by needle or surgical biopsy.

A 165 patient subset of the 510 samples from the five centers had matched plasma and tissue samples. Solid tumor cancer histologies included 57 colorectal cancers (CRC), 22 other GI (non-CRC), 18 melanoma, 18 lung cancer, 15 breast cancer, 8 genitourinary cancer, and 27 other cancer types of lesser frequency. 24% were stage III, 73% stage IV and 3% were unknown. The median age was 58 (range 33–88) and 57% were male and 43% female. Clinical sensitivity for digital sequencing for all mutated oncogenes compared to tissue-based NGS was 85.0%, specificity 99.6%, diagnostic accuracy 99.3%. For tissue-based NGS compared to digital sequencing of cfDNA as the reference, clinical sensitivity was 80.7%, specificity 99.7%, diagnostic accuracy 99.3% (Fig 7A and 7B). Both sample types were processed on an Illumina HiSeq 2500 next generation sequencer.

In addition to the comparison of cfDNA to tissue-based DNA for the whole 54-gene panel, we constructed two-by-two contingency tables on an individual gene-by gene basis for each of the eleven genes with two or more mutated samples. Clinical sensitivity for cfDNA in individual genes ranged from 50% to 100%, and specificity from 79% to 100% as shown in S3 Table. Averaging the performance for each of the eleven most commonly mutated genes using cfDNA resulted in sensitivity of 86.5% (95% CI 80.3%–91.1%), specificity 98.1% (95% CI 97.3%–98.7%) and diagnostic accuracy 97.0% (96.0%–97.7%). Averaging the performance for tissue-based DNA NGS resulted in sensitivity 83.1% (95% CI 76.7%–88.2%), specificity 98.5% (97.8%–99.1%) and diagnostic accuracy 97.0% (95% CI 96.0%–97.7%). Thus, diagnostic performance for the most commonly mutated genes in the 54-gene panel was similar to that for the most commonly mutated genes for both digital sequencing of cfDNA and for tissue-based DNA NGS.

## Discussion

The analytic validation studies of the Guardant360 cfDNA panel highlight its differences from both solid tumor and other cell-free circulating tumor DNA genomic testing methods. The digital sequencing platform enables single molecular sensitivity, an analytic sensitivity that is two orders of magnitude greater than an Illumina HiSeq with standard prep while maintaining near-perfect specificity (Fig 1A and 1B). To overcome the noise in sequencing DNA at the low mutant allele fractions typical of somatic SNVs, near-perfect specificity is required. Whereas other cfDNA NGS methods may claim high specificity, these typically evaluate a single or small number of pre-defined genes, and within those genes just the most frequently mutated hotspots or a “hot exon” [27]. Because the risk of false positive results increases with sequencing of larger targeted regions, an “apples to apples” comparison of different NGS methods to digital sequencing would require evaluation of false positive rates while sequencing many complete exons in many target genes. Since the average gene has a mean of 8.8 exons each a mean of 145 base pairs (bp) long, the average gene has a mean exonal sequence length of ~1,300 bp [40]. Complete exon sequencing for 50 genes thus requires reading lengths of tens of thousands of base pairs. The Guardant360 panel at the time of this study sequences ~78,000 base pairs (78 kbp), providing complete coverage for all exons in 18 genes and the critical exons in the remainder of the 54 total genes. Thus the current analytic validation study finding of zero false positives after sequencing 1.56 million base pairs (78 kbp times 20 patient samples) establishes a new gold standard for analytic specificity relative to other NGS methods. Other NGS liquid biopsy methods such as CAPP-Seq reported 96% specificity and although SafeSeqS reported 99.2% specificity this latter method was only tested on a single gene, *KRAS*, in a single cancer (colorectal) [28,29]. It should be noted that 99% specificity and a 1% false positive rate would generate 780 false positives on a 78,000 bp targeted sequencing region. The achievement of > 99.9999% specificity at the long sequencing target region required for a comprehensive 50-plus gene panel, and at the very low concentrations of somatic SNVs in plasma, is the primary differentiator of this digital sequencing panel versus other cell-free DNA NGS methodologies. The ultra-high specificity enables complete exon sequencing of all or the critical exons in 54 genes, in contrast to multiplex panels of hot-spot tests. The digital sequencing methodology could theoretically be applied to whole exomes or genomes, although panel expansion is expected to be gradual as covered genome alterations should be targetable or enable enrollment in later phase clinical trials, in order to have clinical utility.

The analytic validation studies in this report provide levels of evidence matching or exceeding the EGAPP, NCCN and Nex-StoCT guidelines [30–33]. As a collaborative study using a large panel of well-characterized samples as well as interlaboratory comparison, they achieve EGAPP Level 1 analytic validity evidentiary standards. Clinical validity based on comparison of a large cohort of 165 consecutive matched plasma and tissue samples from five different centers across multiple cancer types also meets EGAPP Level 1 evidentiary standards. Nex-StoCT guidelines establishing NGS test systems for clinical use are achieved via in-depth studies of analytic sensitivity and specificity, assay robustness and interfering substances (genomic DNA), precision including both repeatability and reproducibility, and sample stability relative to storage time and temperature stress.

The clinical sensitivity for plasma-based DNA NGS via digital sequencing was equivalent to the clinical sensitivity of tissue-based DNA NGS sequencing. The approximately 15% false negative rate with cfDNA (and 19.3% rate with tissue-based sequencing) in advanced cancer patients in this study highlights an important clinical limitation: a negative finding (no mutation detected) does not rule out the presence of a potential genomic target with either sample type. Although the cfDNA digital sequencing method has high analytic sensitivity and can detect a single mutated DNA fragment as low as 0.06% MAF, its clinical sensitivity is limited by biology: not all tumors release DNA into circulation. Considering the ultra-high analytic specificity of the digital sequencing method, it is possible that Guardant360 false positives in concordance studies are actually tissue NGS false negatives. It has been previously shown that the discordance between genomic alterations reflected in cfDNA but not in tissue samples tended to be related to spatial and temporal tumor heterogeneity, especially older tissue biopsy material, possibly related to tumor evolution [41].

This study validates the Guardant360 assay across many cancer types, but only in stage III/IV cancers. Analysis of early stage tumors and indolent tumors may have higher false negative rates. Additionally, primary brain tumors may not release DNA into the systemic circulation due to the blood-brain barrier and thus this assay should be specifically validated for this indication in future studies [28]. A further limitation of this study is that individual substudies had small sample sizes, however there were consistent results between the findings of near-perfect analytic specificity (comparison to whole exome) and clinical specificity (comparison to tissue NGS) results, and the clinical sensitivity reported here for cfDNA of 85.0% across 54 genes is comparable to that reported by on a single-gene cfDNA assay of 87.2% [28]. Lastly, although not a limitation of this study, we did not validate the quantitative accuracy of the MAF or gene copy number amplification, instead comparing the binary presence or absence of a single nucleotide variant/point mutation or focal gene amplification.

The Guardant360 panel is an ultra-high specificity test that should be utilized to rule in mutations with high confidence. Although clinical specificity was reported as 99.6% compared to tissue-based NGS as the reference standard, considering that the analytic specificity of digital sequencing is > 99.9999%, it seems likely that a Guardant360 “false positive” in fact reflects a tissue-based NGS “false negative”. Quantitative PCR and other methods may have higher sensitivities than our method that could prove useful in stage II cancers. However, when tissue-based NGS was compared to digital sequencing as the reference standard on sequential samples in this study, clinical sensitivity of tissue testing was also imperfect. One hypothesis for the 19.3% false negative rate for tissue biopsy-based NGS may be that core needle or even surgical biopsy may incompletely capture all the intra- or inter-tumor heterogeneity, whereas plasma may act as a more complete “summary” of the DNA released into circulation from multiple tumor clones within and between lesions. Spatial (both intra- or inter-tumor) heterogeneity has been established in malignancies of the breast, bladder, ovary, prostate, pancreas, kidney, lung and colon [11,41], raising the likelihood of sampling error inherent in tissue biopsy-based NGS methods.

The clinical utility of the Guardant360 diagnostic test in advanced cancer is supported by results in clinical practice. Seventy-six percent of 1,000 consecutively tested patients had a reported somatic single nucleotide variant amongst 54 genes or a copy number amplification in three genes. This was accomplished without an invasive tissue biopsy. Although 86% of stage III/IV patients had positive results, in actual practice the yield was 76%, reflecting that the test was ordered on at least some earlier stage patients, and patients whose ctDNA was suppressed by active chemotherapy or radiation therapy. The percentage of patients with a positive result is expected to increase further as new genes and types of genomic alterations (e.g. more amplifications, fusions, indels) are added. Another “liquid biopsy” method, circulating tumor cells (CTCs), may only be detectable in 30%–50% of patients with metastatic disease [42]. Somatic SNVs have been compared as potential biomarkers across multiple types of advanced cancers and it has been shown that cfDNA is present in higher concentrations, by two orders of magnitude, than elevations in CTCs [43–45]. When present, CTCs are useful prognostically, and independent of clinical risk factors and serum biomarkers, but their utility is limited because CTC counts themselves do not inform as to targeted therapy. In addition, switching chemotherapy choice in the face of elevated CTC counts did not improve overall or even progression-free survival (PFS) in a recent randomized study of metastatic breast cancer [46].

While systemic therapy is increasingly effective, it is rarely curative, and almost all patients with metastatic disease experience eventual progression of their malignancy despite treatment [47]. There are many examples of genomic alterations that necessitate real time re-evaluation of evolving tumors. In colorectal cancer *KRAS* exons 12 and 13 mutations drive de novo or acquired resistance to anti-EGFR therapy [48] and *KRAS* and/or *BRAF* status may change with time between primary and metastatic lesions in colorectal cancer in 17% of patients [49]. In metastatic breast cancer changing HER2 (coded by *ERBB2* gene) status may occur in 24% of initially HER2 positive tumors which escape trastuzumab by mutating to HER2 negative [50]. Resistance to aromatase inhibitors develops in almost half of estrogen receptor (ER) positive breast cancer patients who acquire *ESR1* mutations [51]. Resistance to first-line tyrosine kinase inhibitors (TKIs) in NSCLC is often driven by the development of tumor clones harboring *EGFR* T790M mutations [52]. The evolution of resistance and importance of adaptively managing treatment to changing genomic status has led to recommendation for repeat tissue biopsies when new metastatic and/or recurrent disease occurs in several cancers, including breast [53], non-small cell lung [1], colorectal [54] and melanoma [55]. Unfortunately, invasive needle biopsies of visceral metastases are accompanied by complications including a 1–2% risk of hospitalization for hemorrhage after liver biopsy [56], or a 6.6% or 2.2% risk of hospitalization for pneumothorax with transthoracic or transbronchial needle aspiration, respectively [57]. Obtaining these biopsies in clinical trials is no less risky, where complication rates for research study-mandated intrathoracic and abdominal/pelvic solid organ biopsies were 17.1% and 1.6%, respectively [58]. Inpatients with cancer may be at particular risk of complications from transthoracic needle biopsy, with one study reporting a 21.4% rate of pneumothorax with 36.0% of these requiring a chest tube [59]. The direct costs of invasive biopsy related to NGS of visceral metastatic cancer are also significant: a thoracoscopic biopsy of a lung lesion can exceed $30,000 [60]. Lastly, indirect costs due to complications can compound direct biopsy costs. For example, transthoracic and transbronchial needle aspiration biopsies of lung lesions were recently reported as having a median Medicare (2013 USD) cost of $3,784 but a much higher average cost of $14,634 because the latter was driven up by the 19% rate of adverse events [61].

The clinical utility of cfDNA lies in its ability to non-invasively assess the clonal evolution of cancer, that is, to elucidate the “why” of cancer progression and to potentially identify new druggable targets without a tissue biopsy. Peter Nowell posited almost 40 years ago that “each patient's cancer may require individual specific therapy, and even this may be thwarted by emergence of a genetically variant subline [subclone] resistant to the treatment” [62]. Temporal heterogeneity may occur by linear evolution or by competition between different subclones in the same tumor that compete with each other for dominance during the disease course or under pressure from targeted therapies [11]. Serial testing of multiple myeloma patients found that the initial biopsy genomics did not reveal the tumor subclone that ultimately drove mortality (secondary plasma cell leukemia) [63].

Continued development of novel targeted therapeutic agents matched to specific genetic alterations have extended survival in cancer patients, often dramatically, compared to cytotoxic chemotherapies [2]. In 2010, Von Hoff found genomic alterations in 98% of advanced cancer patients tested utilizing immunohistochemical (IHC) stains and DNA oligonucleotide assays of gene expression in 51 target genes. In this multicenter study of patients who had failed at least two lines of therapy, matching therapy to the genomic findings resulted in an extension of PFS 1.3-fold or longer compared to their previous line of therapy in 27% (95% CI, 17% to 38%) of tested patients [64]. In 2012, Tsimberidou and colleagues performed multiplex testing based primarily on NGS for 13 alterations in 12 genes in 1,144 advanced cancer patients (who had failed on average five lines of therapy), and found 40% with genetic alterations. Although not randomized, matched therapies resulted in significantly higher overall response rate (27% vs. 5%), longer time-to-treatment failure (5.2 vs. 2.2 months), and longer overall survival (median, 13.4 vs. 9.0 months) in 176 tested patients vs. 116 untested controls [65]. In 2014, the same investigators repeated this pan-cancer study in 1,276 patients and found that a higher number, 57.8%, had a genomic alteration, which they attributed to the use of NGS to sequence a broader panel of genes [66]. The matched targeted therapy arm again demonstrated a significantly higher objective response rate, improved overall survival and PFS than with the non-matched therapy.

Regardless of the impact on survival, the clinical utility of this cfDNA panel is self-evident. By obviating the need for an invasive biopsy when the patient’s initial biopsy is QNS for molecular testing, the Guardant360 panel enables biopsy-free multiplex testing of all current guideline-recommended genomic targets without the costs and complications involved in invasive tissue biopsies for NGS. In addition, this cfDNA panel dramatically reduces the total time from initial evaluation to treatment (TTT) of advanced cancer patients, by eliminating the time lost waiting for an invasive biopsy to be scheduled and performed, and for pathological material to be reviewed and qualified before NGS can be conducted [67]. The 99.8% assay success rate for the Guardant360 panel starkly contrasts with tissue-based NGS assay failure rates of about 25% (20% because tissue is unavailable or QNS plus a 5% NGS assay failure rate), sparing repeat invasive biopsies [68]. This figure is comparable to a previous study of a hotspot cfDNA assay for *EGFR* mutation in NSCLC—19% of invasive biopsy tissue samples were unusable because they were QNS or there was some technical problem [69]. As the number of matched therapies and genomic targets grows, serial singleplex testing will increasingly exhaust available tissue for genomic testing, while the utility of this cfDNA NGS approach will continue to rise [8,26]. In difficult to biopsy visceral cancers such as pancreatic and biliary cancers, a recently published prospective study of Guardant360 vs. tissue-based NGS found that the tissue samples were quantity not sufficient (QNS) for genomic testing in over 35% of the patients with stage III/IV cancers. Whereas tissue-based NGS was only positive in 62% of patients, Guardant360 was positive in 85% of patients. In addition, this external validation study showed that 90% of mutations detected in tumor biopsies were also detected in cfDNA and that diagnostic test performance was consistent or better than reported here with cfDNA sensitivity of 92%, specificity 100% and diagnostic accuracy of 98% across five informative genes [70]. A second recently published study using Guardant360 also in advanced pancreatic carcinoma found mutations detected in plasma in 84% of patients and 100% concordance with tissue analyzed for mutations in the *KRAS* gene [71]. These results are consistent with those of the current study and add a high level of evidence for clinical validity because in these two studies Guardant Health was blind to the external laboratories’ results [72].

How should the test be used today? The actionability of a given gene mutation is strongly related to cancer sub-type. Therefore, this biopsy-free NGS panel should not replace the initial tissue biopsy, as thorough histopathological diagnosis of the primary tumor is critical. For example, *ERBB2* (HER2) copy number amplification in NSCLC does not predict TKI response as it does in breast cancer [73]. Similarly, *BRAF* V600E inhibition has an 81% response rate in melanoma patients with advanced disease—but only a 5% response rate in advanced colorectal cancer [74]. However, once the histopathological diagnosis is established, samples from the initial biopsy may be exhausted with insufficient tissue remaining for NCCN-recommended multimarker testing. In these cases, Guardant360 could be used *in lieu* of a repeat invasive biopsy for NGS, particularly for patients with visceral primary disease or metastases to lung, liver or bone. Secondly, in patients with visceral primary or metastatic disease, when a tumor has progressed or recurred based on clinical symptomatology, imaging or serological protein biomarkers, e.g. CEA, CA-125, CA 15–3, PSA, etc., the Guardant360 panel could be used to re-evaluate for new targeted therapies, enabling real-time adaptive management of a patient’s continuously evolving cancer while obviating further invasive tissue biopsies.

## Conclusions

Approximately 90% of cancer-related deaths occur from metastatic disease rather than advanced local disease [75]. However, most targeted cancer therapies are matched to identification of genomic driver mutations in primary tumors. Recognition that metastatic lesions have a genomic fingerprint that may evolve and become discordant from the primary tumor has led to recommendations for invasive biopsies for genomic testing of visceral metastases in several cancers. The studies presented here are the first to provide analytic and clinical validation of the accuracy of a comprehensive tumor profiling test that utilizes NGS of circulating tumor DNA to identify genomic alterations in hundreds of exons in over 50 genes with unparalleled specificity. Digital sequencing relies on massively parallel sequencing of circulating tumor DNA fragments combined with algorithms that utilize multiple analytic inputs to accurately compute quantitative SNV concentrations and absolute gene copy numbers in plasma. This multianalyte algorithm plasma-based assay enables avoidance of the cost, complications, time delay, and failure rates inherent in solid tumor tissue-based NGS. By ending the diagnostic odyssey without an invasive biopsy, this approach demonstrates clinical utility as defined by EGAPP, while accurately identifying a genomic alteration in over three-fourths of tested patients, of which over three-fourths are actionable in a cohort of 1,000 patients in actual clinical practice. Future investigation should evaluate whether increasing the size of the cfDNA gene panel can increase the yield of positive, and actionable, results.

## Materials and Methods

### Characteristics of the Guardant360 panel and clinical diagnostic assay

The Guardant360 cfDNA NGS panel identifies SNVs via sequencing of 54 genes spanning approximately 78,000 base pairs, including complete exon coverage in all exons in 18 genes and the critical exons in 36 genes (gene list in S1 Table) as well as copy number variations (CNVs) in *ERBB2*, *EGFR*, and *MET*. The gene list was selected to focus on those genomic alterations that are currently actionable, which is defined as being targets of sensitivity or resistance to an FDA-approved matched therapy and/or a targeted therapy in later stage clinical trials.

The exons in each gene targeted by the capture probes are listed in S4 Table. The enriched digital sequence libraries are then analyzed using HiSeq 2500 Sequencing System (Illumina) paired-end sequencing with fluorescent reversible terminator deoxyribonucleotides [76]. At the time of this study, the Guardant360 panel targeted region was 78,000 base pairs (78 kbp) per sample and each base was sequenced at average raw coverage depth of 8,000X with a minimum average base coverage of 3,000X and a minimum Qscore of 20.

Two 10mL tubes of whole blood were collected per individual in Streck Cell-Free DNA Blood Collection (Streck) tubes, which contain a proprietary formaldehyde-free preservative that stabilizes white blood cells, preventing the release of genomic DNA and allowing isolation of high-quality cfDNA. It has been previously shown that cfDNA is stable in Streck tubes without release of genomic DNA from leukocytes in whole blood stored at room temperature for seven days or when shipped overnight by air, preventing need for preliminary centrifugation prior to shipping [77]. Model cell lines were cultured according to manufacturer recommendations. The cell-free DNA from these cell lines is extracellular DNA released by the culture cells and the fragmentation pattern is essentially identical to circulating DNA except the size distribution peaks tend to be slightly broader. Similar biological mechanisms to those in play in circulating peripheral blood *in vivo* occur with the cell culture media, i.e. DNA digestion of histone-protected genomic DNA. Plasma samples and supernatant from cultured cell lines were processed using the QIAamp Circulating Nucleic Acid Kit (Qiagen) according to the manufacturer’s instructions. Cell-free DNA is extracted from 1.5 mL to 5 mL plasma and subsequently concentrated and size selected using Agencourt Ampure XP beads (Beckman Coulter) and subsequently quantified using Qubit® 2.0 fluorometer. The DNA is extracted using standard extraction procedures, including proteinase K digestion of histone and other DNA-binding proteins and isoproponal precipitation. The DNA is then end-repaired to repair ends of DNA with a 5’ phosphate prior to ligation of adapters necessary for downstream sequencing. The final volume is 36 μL water and must be at least 0.14 ng/μL (5 ng input) to proceed with library preparation.

Isolated cfDNA fragments are subsequently converted to digital sequence libraries. Briefly, in the digital sequencing workflow, non-unique oligonucleotide heptamer barcodes are ligated to each half of individual double-stranded cfDNA. This generates a duplex library whereby each single-stranded half of the original double-stranded input cfDNA sample is separately encoded with said oligonucleotides (S2 Fig). This digital conversion process occurs with high efficiency with greater than 80% of the original plasma cfDNA molecules converted. This efficiency at 10ng input is at least double the theoretical efficiency of amplicon-based methods (e.g., BEAMing [78], TAM-Seq [27], etc) and at least 5 to greater than 10-fold the reported 3–13% efficiencies of ligation-based (e.g. highly optimized off-the-shelf next-generation sequencing kits [79]) genetic analysis methods. Specifically, theoretical conversion efficiency of amplicon-based methods on cell-free DNA can be approximated by (1-x/166)*100%, where x is the length of the amplicon. For x = 100bp, theoretical efficiency is 39% and drops significantly with higher levels of multiplexing due to inversely lower individual concentrations of specific PCR primer pairs. High efficiency conversion is critical since it translates directly to sensitivity, i.e., one cannot achieve single-molecule sensitivity if only a fraction of a few input DNA molecules are convertible to analyzable material. Consequently, the conversion process results in a self-referenced digital sequence library with properties similar to differential signaling in digital communications. These digital sequence libraries are amplified and then subsequently enriched for target genes using biotinylated custom baits of RNA probes. Input range for the digital sequencing assay was between 5–30 ng (a haploid genome copy has a mass of 3.3 pg, therefore this input range is equivalent to 1,500–9,000 genome copies, i.e., 5 ng/[0.0033 ng/genome copies] or 30 ng/[0.0033 ng/genome copies]).

### Bioinformatics processing of cell-free DNA sequencing data and variant calling

Just as digitization of communications signals in combination with an error model of communication channels enable post-transmission signal processing and error correction, digitization of cfDNA and a complete base-by-base model of sequencing workflow error modes enables post-sequencing removal of false positive errors via bioinformatics processing. Post-sequencing, the digital decoding process comprises analysis of both strands of each unique cfDNA molecule to greatly increase the accuracy (“transmission characteristics”) of each individual base call. This process is akin to building a separate noise model for each and every one of the 78,000 bases in the sequencing panel. Each strand of a double-stranded cfDNA molecule is individually tagged, allowing custom software to compare the two complementary strands to ascertain whether either has acquired an erroneous variant due to a sequencing error, library preparation error, or DNA damage during sample processing. Additionally, the minor base frequencies from a set of normal samples serve as a baseline for all sites in the panel of targeted regions, and inform the per-base noise models. The variants for a given sample are measured against that baseline and positive variant calls must achieve a signal level above each specific base’s noise baseline.

Software utilized includes CASAVA (version 1.8.4), the open source BWA-MEM aligner, and a custom read pile-up process that utilizes information encoded by digital-sequencing oligonucleotides to reconstruct the set of unique cfDNA molecules. First, 150bp paired-end reads are aligned to the reference genome. Trim_galore, a wrapper script for cutadapt, is used to remove lower quality 3’ adapter sequences, and custom scripts are used to remove unaligned sequences and trim low quality tails and adapter contaminations. Custom scripts are then used to (a) remove spurious variants (“noise”) created by sequencing errors, (b) identify all germline single nucleotide polymorphisms (SNPs) and somatic single nucleotide variants (SNVs), and (c) call somatic SNVs, while removing erroneous variants resulting from sequencing errors, DNA damage, strand bias, etc. These steps complete the digital sequencing process and enable post-sequencing removal of the false positives associated with detecting somatic variants present in cfDNA at low concentrations. The fractional concentration or mutant allele fraction for a given mutation is calculated as the fraction of circulating tumor DNA harboring that mutation in a background of wild-type cfDNA fragments at the same nucleotide position. Because digital sequencing enables tracking and quantification of all unique cfDNA fragments overlapping a given genomic site, PCR duplicates and artifacts of amplification during library preparation can easily be removed.

Leveraging the ability of digital sequencing technology (DST) to absolutely quantify the number of unique DNA fragments in a sequenced sample, the copy number of a given gene in plasma may also be ascertained. To determine the CNV or amplification of a given gene, we first measure the total number of unique fragments covering each gene comprised of both halves of the original parent molecules. Regions with copy-number alteration bias will have uneven representation of each half of the original double-stranded library. The deviation from ideal distribution is calculated and modeled through a non-parametric regression to remove sample-preparation- and sequencing-associated biases. Subsequently, the mode of the normalized number of fragments covering each gene is calculated to estimate the fragment number corresponding to two copies to derive a baseline diploid value. All values of unique fragments for each gene are then normalized by this baseline value. The baseline derivation is informed by molecule counts data from a large set of normal samples from healthy donors’ plasma (Normal Set) provided by AllCells (Alameda, CA) with donor consent. The age range of donors is 20 to 40 years and input plasma is screened to provide a minimum input of 15 ng of cfDNA. A statistical z-score for each gene is calculated by comparing the normalized gene fragment number to that derived from the Normal Set to estimate the likelihood of the gene exhibiting amplification and remove any remaining region specific bias. Gene amplifications with a z-score > 2.5758 (the inverse of the cumulative distribution function of a Gaussian distribution at 99.5% confidence level) are called as truly amplified. The numerical result of the above procedure expresses the absolute copy number of a gene in plasma-derived cfDNA samples, which is a combination of both normal cfDNA (mainly leukocyte-derived) and tumor-derived gene copy number. Because most of the cfDNA is typically germline-derived, a small elevation in the gene copy number in plasma may reflect a much higher copy number in the tumor. For example, if the *ERBB2* gene copy number in the tumor was 10.0, and 5% of the gene in cfDNA was tumor-derived and 95% was leukocyte-derived (germline copy number 2.0), then the *ERBB2* copy number in plasma would be 2.4. Thus, the Guardant360 panel expresses the absolute copy number or focal amplification of a gene in the plasma, and when 2.2 or higher, this may represent a clinically significant increase in the copy number of the gene in cancer cells in tumor samples.

### Accuracy and Analytic Specificity for SNVs

Nex-StoCT defines accuracy as the degree of agreement between the nucleic acid sequences derived from the assay and a reference sequence. It defines analytic specificity as the probability that the assay will not detect a sequence variation when none are present (the false positive rate is a useful measurement for sequencing assays). As a reference standard, 2 micrograms of genomic DNA were isolated from leukocytes (WBCs) from 20 patient whole blood samples for whole exon sequencing at an outside reference laboratory (Ambry Genetics, Aliso Viejo, California). An aliquot of cfDNA extracted from the same matched blood sample was spiked (referred to as spike-in) into a normal control sample (referred to as matrix) at ~5% concentration, which is equivalent to ~2.5% MAF for heterozygous SNVs and ~5.0% MAF for homozygous SNVs. A total of 10 ng cfDNA, including the spike-in and matrix underwent digital sequencing.

To determine the true germline nucleotide identity of position chr11:534,242 (*HRAS*) in the putative false positive sample, gDNA was extracted from blood using the QIAamp DNA Blood kit and sent for Sanger sequencing at the Stanford Protein and Nucleic Acid Facility (Palo Alto, CA). A total of four reactions were performed, two with a forward primer and two with a reverse primer. For each of the four reactions 10 μL of a 150 ng/μL gDNA stock were used with primers (5 μM) sent separately for both forward and reverse reactions. The primer sets (Primer ID Hs00326065_CE, Life Technologies) were designed to amplify a 509 bp region in *HRAS* at chr11:532242–535550. The forward primer (CTCTAGAGGAAGCAGGAGACAGG) binds at position chr11:532242–535550 and the reverse primer (CATCACTGGGTCATTAAGAGCAA) binds at position chr11:534081–534589. Traces were inspected manually (FinchTV) to confirm the base identity at chr11:534,242. All four sequencing traces show an equal intensity peak corresponding to A and G nucleotides at this position indicating a heterozygous germline SNP in *HRAS* (H27H), consistent with the digital sequencing data.

### Analytic Sensitivity for SNVs

Analytic sensitivity is defined by determining the lowest concentration or amount of the analyte or substance that can be measured with at least 80% probability, reflected as limit of detection. A spiked dilution series of one normal cfDNA sample into another normal cfDNA sample with total input material totaling 30 ng was created and repeated twice for a total of three replicates. Dilutions for each sample at 30%, 10%, 1%, 0.5%, 0.3% and 0.1% titrations were prepared and sequenced in separate batches on separate days.

### Precision for SNVs

Repeatability and reproducibility according to Nex-StoCT and New York State Department of Health (NYSDOH) guidelines on samples mimicking patient samples (ie spike ins of either cfDNA from healthy plasma donors or cell line media). Nex-StoCT defines precision as the degree to which repeated sequence analyses give the same result repeatability (within-run precision) and reproducibility (between-run precision). Repeatability was assessed via comparison of replicate sequencing of 5 samples used in the accuracy study with the same lot, operator and sequencer, and reproducibility was assessed via sequencing of 5 samples used in the accuracy study with different reagent lots, different operator and at least one week following initiation of the experiment.

### Analytic Sensitivity and Specificity of Copy Number Variation

Similar methods were used to assess accuracy, analytic specificity and sensitivity (limit of detection), and assay precision for the absolute copy number of a given gene in circulating cfDNA. Specifically, for analytic sensitivity of CNVs, limit of detection (LOD) was determined by creating a pool of three separate cell lines at various levels of CNVs. The three cell lines combined for the LOD pools were AU565, HCC827, and H1993 (ATCC). The LOD was performed at three different input amounts, 5, 15, and 30 ng, and in duplicate. The LOD pools were made to approximately contain amplifications of 200% (4 copies), 100% (3 copies), 60% (2.6 copies), 30% (2.3 copies), 20% (2.2 copies), and 10% (2.1 copies) for each gene tested in the assay: *EGFR*, *ERBB2* and *MET*.

### Assay Robustness

As genomic DNA from lysed leukocytes could potentially interfere with the assay, the “worst-case scenario” for sample stability and contaminating leukocyte DNA was assessed. Ten plasma samples were split in half, and one set was cycled at 37°C for 8 hours per day for five days and the other set was processed immediately upon receipt (two days shipping) without temperature cycling. Comparisons of these split patient samples showed near perfect concordance in the variants detected (Fig 5B).

### Patient samples

Institutional Review Board (IRB) review was obtained and informed consent was obtained at the five centers (Cancer Centers of Excellence, John Wayne Cancer Institute, MD Anderson Cancer Center, Samsung Medical Center, and University of California San Francisco) contributing 510 plasma and 165 matched tissue samples from stage III/IV cancer patients. All NGS results from the 165 paired plasma and tissue samples are filed in a public data repository in Data Dryad at http://datadryad.org. The 1,000 samples analyzed in the CLIA laboratory for clinical purposes were de-identified/anonymized for the observational analysis.

### Clinical Validity

For clinical validity, 510 consecutive plasma samples were collected from the five different institutions in the United States and Korea. Of these 165 concurrent tissue biopsy samples were collected. The 165 matched plasma and tissue samples were both analyzed on the Illumina Hi-Seq 2500 platform, the cfDNA analyzed using the digital sequencing method described herein and the DNA isolated from tissue samples analyzed using standard prep as per the manufacturer’s instructions [76]. Contingency tables were constructed to calculate sensitivity, specificity and diagnostic accuracy gene-by-gene as well as for the whole 54-gene panel and confidence intervals by standard methods [80,81].

## Supporting Information

S1 Fig Analytical Sensitivity of Guardant360 for detecting 6 copies of *ERBB2* (HER2), *EGFR* or *MET*. The Guardant360 test confidently detects amplifications of 6 copies within a circulating tumor fraction greater than 2% for *EGFR* and *MET* and greater than 10% for *ERBB2*. At tumor fractions below these values, p-values become greater than 0.01. A copy number of six in the tumor correlates to an IHC HER2 3+ and exceeds a FISH ratio of HER2 or MET to CEP17 of > 2.2.
(EPS)

S2 Fig Schematic of Digital Sequencing Technology. After isolation from plasma, each single-stranded half of the original double-stranded 5–30 ng input cfDNA sample is separately encoded with oligonucleotide heptamers to create a self-referenced digital sequence duplex library with properties similar to differential signaling in digital communications. These digital sequence libraries are amplified and enriched for target genes using biotinylated custom baits of RNA probes. The enriched digital sequence libraries are then analyzed using HiSeq 2500 Sequencing System (Illumina) paired-end sequencing with fluorescent reversible terminator deoxyribonucleotides and referenced for 54 genes requiring a 78,000 base pair targeted region. Average raw coverage depth of 8,000X. Just as digitization of communications signals enables post-transmission signal processing and error correction, digitization of cfDNA enables post-sequencing removal of false positive errors via heavy bioinformatics.
(EPS)

S1 Table Guardant360 54-gene cell-free DNA NGS panel. Genes in bold with **complete exon** vs. critical exon coverage.
(DOCX)

S2 Table Detail for concordance study of 165 matched plasma vs. tumor sample DNA NGS. Somatic cancer variants from 135 patients with matched tumor DNA sequencing and plasma cell-free DNA (cfDNA) sequencing. Thirty additional patients had no somatic cancer variants detected in either tumor or blood. Definitions of column headers are as follows: **refbase**, reference genome (hg19) base at the position of genetic variant; **mutbase**, variant base; **blood_acc**, Guardant Health sample accession number for plasma cfDNA; **t_acc**, Guardant Health sample accession number for tumor DNA; **blood_VAF**, Variant Allele Frequency in cfDNA; **t_VAF**, Variant Allele Frequency in tumor DNA; **concordance**, whether variant was detected only in tumor DNA (“tumor”), only in plasma cfDNA (“blood”), or in both (“conc”); **tumor_detected**, whether tumor was detected in plasma cfDNA. The percent-agreement calculation was performed on samples where tumor was detected in cfDNA (“Y”).
(XLSX)

S3 Table Two-by-two contingency tables for individual genes for the eleven most commonly mutated genes in the 54-gene panel as well as for the entire panel as a whole. The first worksheet compares cfDNA results against tissue NGS as the reference standard and the second worksheet compares tissue NGS against cfDNA NGS as the reference standard. Sensitivity, specificity and diagnostic accuracy with 95% confidence intervals are calculated for the eleven genes averaged together. Then the results using 2 x 2 tables for the entire 54-gene panel are calculated at bottom of each spreadsheet with 95% confidence intervals.
(XLSX)

S4 Table Covered exons in each gene in the 54-gene panel. (XLSX)
