# Supplementary material for: Analytical and Clinical Validation of a Digital Sequencing Panel for Quantitative, Highly Accurate Evaluation of Cell-Free Circulating Tumor DNA
Source: PLoS One. 2015 Oct 16;10(10):e0140712. doi: 10.1371/journal.pone.0140712 (PMC4608804; doi:10.1371/journal.pone.0140712)
Supplement: S1 Table — Genes in bold with complete exon vs. critical exon coverage. (DOCX) [file pone.0140712.s003.docx]

| *ABL1* | *AKT1* | ***ALK*** | ***APC*** | ***AR*** | *ATM* |
| --- | --- | --- | --- | --- | --- |
| ***BRAF*** | *CDH1* | ***CDKN2A*** | *CSF1R* | *CTBBB1* | ***EGFR****** |
| ***ERBB2****** | *ERBB4* | *EZH2* | ***FBXW7*** | *FGFR1* | *FGFR2* |
| *FGFR3* | *FLT3* | *GNA11* | *GNAQ* | *GNAS* | *HNF1A* |
| *HRAS* | *IDH1* | *IDH2* | *JAK2* | *JAK3* | *KDR* |
| *KIT* | ***KRAS*** | ***MET****** | *MLH1* | *MPL* | ***MYC*** |
| ***NOTCH1*** | *NPM1* | ***NRAS*** | *PDGFRA* | ***PIK3CA*** | *PTPN11* |
| ***PTEN*** | ***PROC*** | ***RB1*** | *RET* | *SMAD4* | *SMARCB1* |
| *SMO* | *SRC* | *STK11* | *TERT* | ***TP53*** | *VHL* |

*Copy number amplification of *EGFR*, *ERBB2* (HER2) and *MET* genes.
